# Syllable-PBWT for space-efficient haplotype long-match query

**DOI:** 10.1093/bioinformatics/btac734

**Published:** 2022-11-28

**Authors:** Victor Wang, Ardalan Naseri, Shaojie Zhang, Degui Zhi

**Affiliations:** School of Biomedical Informatics, University of Texas Health Science Center at Houston, Houston, TX 77030, USA; School of Biomedical Informatics, University of Texas Health Science Center at Houston, Houston, TX 77030, USA; Department of Computer Science, University of Central Florida, Orlando, FL 32816, USA; School of Biomedical Informatics, University of Texas Health Science Center at Houston, Houston, TX 77030, USA

## Abstract

**Motivation:**

The positional Burrows–Wheeler transform (PBWT) has led to tremendous strides in haplotype matching on biobank-scale data. For genetic genealogical search, PBWT-based methods have optimized the asymptotic runtime of finding long matches between a query haplotype and a predefined panel of haplotypes. However, to enable fast query searches, the full-sized panel and PBWT data structures must be kept in memory, preventing existing algorithms from scaling up to modern biobank panels consisting of millions of haplotypes. In this work, we propose a space-efficient variation of PBWT named Syllable-PBWT, which divides every haplotype into syllables, builds the PBWT positional prefix arrays on the compressed syllabic panel, and leverages the polynomial rolling hash function for positional substring comparison. With the Syllable-PBWT data structures, we then present a long match query algorithm named Syllable-Query.

**Results:**

Compared to the most time- and space-efficient previously published solution to the long match query problem, Syllable-Query reduced the memory use by a factor of over 100 on both the UK Biobank genotype data and the 1000 Genomes Project sequence data. Surprisingly, the smaller size of our syllabic data structures allows for more efficient iteration and CPU cache usage, granting Syllable-Query even faster runtime than existing solutions.

**Availability and implementation:**

https://github.com/ZhiGroup/Syllable-PBWT

**Supplementary information:**

Supplementary data are available at *Bioinformatics* online.

## 1 Introduction

Developments in genotyping technologies have accelerated the growth of genotype databases, paving the way for systematically comparing the haplotype sequences inherited by individuals (Campbell *et al.*, 2015; Nielsen *et al.*, 2011). Long shared DNA segments, known as Identical by Descent (IBD) segments, between the haplotypes of two or more individuals are highly indicative of a recent common ancestor (Thompson, 2013). To efficiently analyze large panels of haplotypes, Durbin proposed the positional Burrows–Wheeler transform (PBWT) (Durbin, 2014), a data structure that reorders haplotypes at every site (i.e. position within a haplotype) to concisely represent local substring matches within sets of aligned haplotypes, and has a construction runtime proportional to the size of the panel. Beyond IBD segment detection (Freyman *et al.*, 2020; Naseri *et al.*, 2019c; Zhou *et al.*, 2020), PBWT has found applications in genotype imputation (Loh *et al.*, 2016; Rubinacci *et al.*, 2020) and studying recombination events (Naseri *et al.*, 2021).

PBWT algorithms come in two flavors: finding all within-panel pairwise haplotype matches (all-vs-all matching), or finding all pairwise haplotype matches between an out-of-panel haplotype and any in-panel haplotype (one-vs-all query). In this work, we are concerned with the one-vs-all query problem, of which an important application is genealogical search. Durbin’s Algorithm 5 (Durbin, 2014) is able to find all set-maximal matches from a query haplotype to any panel haplotype, where a set-maximal match is said to exist from a haplotype *s*_1_ to a haplotype *s*_2_ if no other haplotype in the panel has a longer match with *s*_1_ that completely contains the range of sites over which *s*_1_ and *s*_2_ match.

However, as noted by Naseri *et al.* (2019a), reporting only set-maximal matches is likely to exclude a lot of valuable match information, since many considerably long matches would not be reported simply because they were overshadowed by a longer match. (Note too that the quality of being set-maximal is not necessarily symmetric; i.e. that a match is set-maximal from *s*_1_ to *s*_2_ does not imply that it is set-maximal from *s*_2_ to *s*_1_, which is unintuitive for genealogical search.) Instead, setting a match length cutoff is more theoretically justifiable and has been the common practice in real-world genealogical search deployed by direct-to-consumer (DTC) genetics companies. In spite of the occasional errors present in haplotype data, DTC genetics companies and other researchers have demonstrated the efficacy of using long matches to determine genealogical relationships (23andMe, 2021; Naseri *et al.*, 2019a; Roberts *et al.*, 2011). In the PBWT-Query work, Naseri *et al.* (2019a) defined an *L*-long match (abbreviated to ‘long match’) to be a match spanning at least *L* sites (or, for genetic distance, at least *L* cM) and presented an algorithm to find all long matches between a query haplotype and a panel in average-case O(N+c) time, where there are *N* sites and *c* reported matches. Remarkably, since *O*(*N*) time is indispensable to read in the query haplotype, and *O*(*c*) to output matches, O(N+c) is the fastest time complexity theoretically achievable.

However, existing PBWT query algorithms are not space-efficient. Although all-vs-all PBWT matching consumes minimal memory as the scanning algorithms only store data relevant to the current site, one-vs-all PBWT query entails retaining data for all sites in memory to enable pointer lookups that are independent in runtime from the number of haplotypes. To bypass previously visited matches and achieve efficient runtime, Naseri *et al.* (2019a) introduced data structures called LEAP arrays, which increase the memory burden on top of what is already required by the original PBWT data structures. To lighten memory usage, Sanaullah *et al.* (2021) developed Algorithms 3 and 4 of d-PBWT, which solve the long match query problem without LEAP arrays in worst-case and average-case runtimes, respectively, of O(N+c). Despite the memory improvement, storing PBWT data structures in memory for the whole genome remains a bottleneck for potential applications, such as online whole-genome query services. For example, to query on the 22 autosomal chromosomes from UK Biobank consisting of 974 818 haplotypes and 658 720 markers, Algorithms 3 and 4 of Sanaullah *et al.* (2021) require 10.1 TB of memory. Accommodating memory usage of this magnitude demands dedicating expensive servers with massive amounts of RAM. Moreover, even the size of UK Biobank’s database pales in comparison to the tens of millions of genotype samples collected by DTC companies, and this number is only set to rise (Khan and Mittelman, 2018).

For servers with relatively limited memory, current alternatives include keeping data on the solid-state drive (SSD) or hard disk drive (HDD), often in tandem with memory-mapped files. However, accessing these sources is accompanied by a significant runtime overhead, which, when memory-mapped files are used, also heavily depends on the similarity between previous and subsequent queries, as discussed by the authors of PBWT-Query (Naseri *et al.*, 2019a). Alternatively, distributing the PBWT panel into multiple servers may lower the memory footprint for individual servers but at the incurred cost of synchronization.

In this work, we present a space-efficient variation of the PBWT data structure, named Syllable-PBWT, with which we in turn present Syllable-Query, an algorithm that solves the *L*-long match query problem with more optimal memory usage and runtime than existing algorithms. One theoretical contribution featured in this work is the replacement of the divergence array, which in past works has gone hand in hand with the PBWT data structure, with polynomial hashing. While the basic idea of chunking into syllables is core to our approach, the innovation mainly lies in our adaptation of PBWT algorithms, which traditionally were geared toward bi-allelic (or at best multi-allelic) sequences, to function on general sequences.

## 2 Materials and methods

### 2.1 Overview

The existing algorithms for the *L*-long match query problem, as presented by Naseri *et al.* (2019a) and Sanaullah *et al.* (2021), use the binary haplotype sequences to construct the PBWT, which we refer to as bit-PBWT. To query with bit-PBWT, said algorithms maintain four full-panel-sized (comprising *MN* integers) data structures: the positional prefix arrays *a*, the divergence arrays *d* and the virtual extension arrays *u* and *v*. We reasoned that the dense encoding by bit-PBWT would be redundant for identifying *L*-long matches for large *L*, since short matches could simply be skipped in applications like genealogical search and association analysis. Thus, we propose Syllable-PBWT, which treats every *B* contiguous sites as one syllable (where B≤⌈L/2⌉; see Supplementary Appendix S5.1) and builds data structures for only every syllable rather than for every site, a technique that can be loosely likened to others in bioinformatics (Ekim *et al.*, 2021). In doing so, Syllable-PBWT reduces the size of positional prefix arrays by a factor of *B*. Further, Syllable-PBWT introduces prefix hash arrays to replace the divergence arrays and virtual insertion arrays. To further reduce the panel size, we perform coordinate compression and build dictionaries at each syllable, leveraging the linkage disequilibrium of the haplotype sequences to collapse the panel until the discovered coarse matches must be restored to site-level detail using the dictionaries. Overall, the space usage of Syllable-PBWT is about *B* times smaller than that of bit-PBWT, as outlined in Table 1. In order to identify all *L*-long matches, we develop the Syllable-Query algorithm using the Syllable-PBWT data structure. The following subsections elaborate upon the presented algorithms and their correctness.

**Table 1.** Space comparison between bit-PBWT and Syllable-PBWT data structures used to query in Algorithm 3 of Sanaullah *et al.* (2021) and Syllable-Query, respectively

Space usage		Bit-PBWT	Syllable-PBWT
Panel of sequences	$\dot{X}/X$	$\frac{1}{32}$	$\frac{1}{B}$
Syllable dictionaries	*r*	—	$\frac{1}{32\rho }$
Positional prefix arrays	*a*	1	$\frac{1}{B}$
Prefix hash arrays	*h*	—	$\frac{2}{B}$
Divergence arrays	*d*	1	—
Virtual extension arrays	*u*, *v*	2	—
Total space		$4+\frac{1}{32}$	$\frac{4}{B}+\frac{1}{32\rho }$

*Note*: Values are in units of *MN* 32-bit integers, where there are *M* haplotypes with *N* sites each (assume *N* is a multiple of *B*). ρ≥1 is defined in Section 2.3.1. The hashes are stored as 64-bit integers, hence the $\frac{2}{B}$ memory from *h*.

### 2.2 Notation

The data we are dealing with is a haplotype panel consisting of aligned binary haplotype sequences. In a sequence *s* (with positions indexed starting from 0), s[b] denotes the value at position *b*, and s[b,e) denotes the sequence of values from position *b* to position *e −* 1, inclusive. An *L*-long match (abbreviated to ‘match’) between sequences *s*_1_ and *s*_2_ is said to start at *b* and end at *e* if $s_{1}[b,e)=s_{2}[b,e),\;s_{1}[b-1]\neq s_{2}[b-1]$ (or *b *=* *0), $s_{1}[e]\neq s_{2}[e]$ (or *e* is the length of the sequences), and e−b≥L for some specified *L*. Let $\dot{X}=({\dot{x}}_{0},\ldots ,{\dot{x}}_{M-1})$ be the panel of *M* haplotype sequences, each with *N* sites, with which queries are to be matched. Off of the haplotype panel $\dot{X}$, we will construct a raw syllabic panel $\bar{X}=({\bar{x}}_{0},\ldots ,{\bar{x}}_{M-1})$ and, in turn, a (compressed) syllabic panel $X=(x_{0},\ldots ,x_{M-1})$. The construction and the length *n* of every raw/compressed syllabic sequence is later described. For any collection of sequences $C=(c_{0},\ldots ,c_{M-1})$ and any position *k*, we define C[k] as $\left(c_{0}[k],\ldots ,c_{M-1}[k]\right)$.

### 2.3 Syllable-PBWT

The Syllable-PBWT data structure consists of the syllabic panel *X* with dictionaries *r*, the positional prefix arrays *a* and the polynomial prefix hash arrays *h*.

#### 2.3.1 Syllabic panel

To shorten the length of the sequences, we split the panel into syllables of *B* sites each, padding the ends of the haplotypes with 0s as necessary. For the *k*th *B*-site syllable of the *i*th haplotype, we parse the binary allele values spanning the *B* sites, i.e. the reverse of ${\dot{x}}_{i}[kB,(k+1)B)$, as a binary number, whose value we assign to ${\bar{x}}_{i}[k]$, syllable *k* of the raw syllabic sequence ${\bar{x}}_{i}\in \bar{X}$. Constructing $\bar{X}$ takes *O*(*MN*) time since it is computationally equivalent to reading in the panel.

**Fig. 1.**
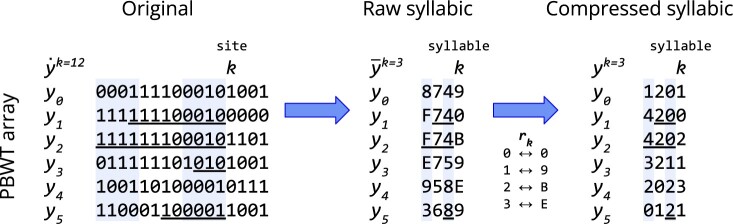
The reverse of every *B *=* *4 binary allele values is parsed as a binary number to obtain raw syllable values, written in hexadecimal (A=10, …, F=15), which undergo coordinate compression to produce the compressed syllable values. Underlines indicate the reverse prefix match before site/syllable *k* with the preceding sequence in the positional prefix order *a_k_*. Although the panel compression appears lossy, the dictionaries *r_k_* can serve to recover the site-level data

Although we have reduced the length of the sequences by a factor of *B* to get $n=\left\lceil \frac{N}{B}\right\rceil$ syllables, our raw syllabic panel $\bar{X}$ still contains the same underlying information, merely arranged into *B*-bit integers, as $\dot{X}$. To reduce the space required to store our syllabic sequences, we observe that the number of distinct raw syllable values at a given syllable is bounded by the number of haplotypes *M*. If $M\ll 2^{B}$, we can apply coordinate compression (i.e. mapping sparse values in a large space to dense values in a small space) to the raw syllable values at a given syllable to obtain the compressed syllable values (abbreviated to ‘syllable values’). To enable conversion between raw and compressed syllable values, we build *r_k_*, a sorted dictionary of the distinct raw syllable values at syllable *k*. Then, every (compressed) syllabic sequence $x_{i}\in X$ can be built as follows: $x_{i}[k]$ is the index of ${\bar{x}}_{i}[k]$ in *r_k_*, where said index can be found with binary search. The second step of Figure 1 illustrates the compression. The raw syllable values can later be recovered using the dictionary: ${\bar{x}}_{i}[k]=r_{k}[x_{i}[k]].$

Since $\bar{X}[k]$ can be written in terms of *r_k_* and X[k], we can avoid the redundancy of keeping $\bar{X}[k]$ in memory after precomputation on syllable *k*. Instead, we store *X* with *O*(*Mn*) memory and *r* with O(B|r|) memory. In the worst case, in which at every syllable, all the sequences have distinct syllable values, then |r|=Mn implies *r* will require O(MnB)=O(MN) memory. Fortunately, in genetic data, linkage disequilibrium [i.e. non-random association of alleles across sites (Slatkin, 2008)] gives rise to repetitive syllable values at any given syllable. Therefore, the ratio $\rho =\frac{Mn}{\left|r\right|}$ will likely be considerable, and *r* will use $O(\frac{MN}{\rho })$ memory. Due to the sorting and binary search on the raw syllable values to compute *r* and *X*, respectively, they each take O(Mnβ log M) time to compute, for some small factor β∈O(B); due to the efficiency of 64-bit architectures, β≪B (see Supplementary Appendix S5.1 for details).

#### 2.3.2 Positional prefix array and PBWT array

The positional prefix array *a_k_* serves as the backbone of PBWT by storing the ordering of the sequences’ reverse prefixes before position *k*. In other words, for the syllabic panel *X*, the position of *i* in *a_k_* is the rank of the reverse of $x_{i}[0,k)$ when sorted (in lexicographical order) among, for all *j*, the reverse of $x_{j}[0,k)$. To simplify notation, the PBWT array *y^k^* is defined such that $y_{i}^{k}=x_{a_{k}[i]}$, i.e. the sequence at position *i* in *a_k_* ($\dot{y}$ and $\bar{y}$ are similarly defined according to $\dot{X}$ and $\bar{X}$, respectively); the *y* arrays need not be kept in memory as they can be expressed in terms of *X* and *a*. Algorithm 1 of Durbin’s PBWT makes use of the binary nature of allele values in bit-PBWT so that two pointers can be used to build $a_{k+1}$ in *O*(*M*) time, given *a_k_* and $\dot{X}[k]$.

To build $a_{k+1}$ for sequences with up to *M* possible syllable values in Syllable-PBWT, we employ similar reasoning to that in bit-PBWT. Figure 1 visualizes the syllabic PBWT array in relation to the binary PBWT array. X[k] is the most significant syllable in determining the ordering of $a_{k+1}$. If two sequences have the same value at syllable *k*, then the tie is to be broken with their reverse prefixes over syllables [0,k). In other words, $a_{k+1}$ can be calculated by sorting the sequences by their X[k] value and, for ties, retaining the ordering from *a_k_*. This can be accomplished in *O*(*M*) time and memory with counting sort, a stable sorting algorithm, since the syllable values are bounded by *M*. Therefore, the positional prefix arrays over the *n* syllables require *O*(*Mn*) time and memory to compute and store.

#### 2.3.3 Polynomial prefix hash function and array for substring matching

The polynomial rolling hash function (Karp and Rabin, 1987) is a simple and efficient hash function for substring matching and has seen use in bioinformatics (Chikhi *et al.*, 2021). One of our main observations is that the divergence arrays are not the only efficient bookkeeping method for positional substring matching in PBWT. The polynomial rolling hash function too can efficiently check if a pair of aligned sequences match over an interval. Specifically, the polynomial rolling hash function of the first *k* elements of *x_i_* is defined as

$$h(x_{i},k)=\left(\sum_{0\leq j<k}\left(x_{i}[j]+1\right)\cdot \mathrm{BAS}E^{j}\right)\;\mathrm{mod}\;\mathrm{MOD}$$

where *BASE* and *MOD* are distinct large primes (Alomair *et al.*, 2010; Karp and Rabin, 1987). In other words, we add 1 to every syllable value, parse the reverse of the resulting $x_{i}[0,k)$ as a number in base *BASE*, and confine it to the range [0,MOD). The benefit of this hash function is that the hash value for any positional substring $x_{i}[j,k)$ can be calculated by $h(x_{i},[j,k))=h(x_{i},k)-h(x_{i},j)\;\;\mathrm{mod}\;\;\mathrm{MOD}$. Thus, the polynomial rolling hash enables efficient substring matching given the prefix hashes. For justification on the dependability of our hash function despite possible collisions (<$10^{-9}$ probability of collision over 10^10^ lookups), see Supplementary Appendix S5.2. For notation, we define the polynomial prefix hash array *h_i_* such that $h_{i}[k]=h(x_{i},k)$ and $h_{i}[j,k)=h(x_{i},[j,k))$.

Every syllable value is added to exactly one prefix hash exactly once, since every hash can simply build off of the previous syllable’s hash. Therefore, the arrays *h* require *O*(*Mn*) time and memory to compute and store.

### 2.4 Syllable-Query

Using the Syllable-PBWT data structures described above (*X*, *r*, *a*, *h*), we present the Syllable-Query algorithm to find long matches between a query haplotype and the panel. Crucial to Syllable-Query will be the hash arrays *h*, designed to replace the data structures *d*, *u*, *v* used for virtual insertion and finding matches. For the binary query haplotype sequence $\dot{z}$, we let $\bar{z}$ be its raw syllabic sequence and *z* be its (compressed) syllabic sequence. In the case that z¯[k]∈rk, we let z[k] be a value distinct from the other X[k] values, such as the size of *r_k_*. We define *h_M_* by the same hash function as above according to *z*. Similarly to before, after reading in the query haplotype in *O*(*N*) time, these sequences require O(nβ log M) time to compute.

#### 2.4.1 Virtual insertion of query haplotype into panel without *u* and *v*

Out-of-panel query is facilitated by virtually inserting the query haplotype into the panel (Naseri *et al.*, 2019a). The virtual locations of the query sequence are stored in an array *t* such that *t_k_* is the position in *a_k_* in which the query sequence would be, had it been included in the original panel. To calculate *t_k_*, past solutions utilize the precomputed auxiliary arrays *u* and *v* at every site *k* to facilitate computing $t_{k+1}$ based on $\dot{z}[k]$ and *t_k_*, where $u_{k}[i]$ is the number of 0≤j<i for which ${\dot{y}}_{j}^{k}[k]=0$, and $v_{k}[i]$ is $u_{k}[M]$ plus the number of 0≤j<i for which ${\dot{y}}_{j}^{k}[k]=1$. Specifically, $t_{k+1}$ is $u_{k}[t_{k}]$ if $\dot{z}[k]=0$ and $v_{k}[t_{k}]$ otherwise. However, past solutions require binary sequences, and the notions of *u* and *v* do not efficiently generalize to *M* possible syllable values.

To find the value of $t_{k+1}$, we binary search among the in-panel sequences for where *z* belongs. To compare *z* with another sequence *x_i_* in one step of the sequence-wise binary search, we first compare their values at syllable *k*, and if they are equal, we binary search for the maximum *b *<* k* for which $z[b]\neq x_{i}[b]$ to compare *z* and *x_i_*. Once again, these fast comparisons are enabled by our hash arrays *h*. Our worst-case time complexity for virtual insertion over the *n* syllables is O(n log M log n), since we binary search over *M* sequences and *n* syllables. In the average case, the O(log n) time binary search would only occur in the small proportion of comparisons for which $z[k]=x_{i}[k]$ (the expected number of such comparisons is *ρ*), and the range of the syllable-wise binary search can be minimized by setting its lower bound to the greatest value of *b* when *t_k_* was being computed, since it is impossible for the start of the longest reverse prefix match with *z* at syllable *k *+* *1 to be earlier than that at syllable *k*.

#### 2.4.2 Identifying long matches virtually near *z*

We define *l* as the minimum number of full syllables within any *L*-site match. To derive the expression for *l*, we must consider the case in which a match extends far into a syllable without completely covering it, i.e. *B −* 1 out of the *B* sites. If the remaining L−(B−1) sites are to minimize the number of full syllables covered, they would not complete the nearly filled syllable but rather extend in the opposite direction. The number of full syllables covered would then be $l=\left\lfloor \frac{L-B+1}{B}\right\rfloor$. We consider matches spanning *l* syllables to be potential long matches, which we will abbreviate to ‘long matches’ or ‘matches’ with the implication that only matches spanning *L* sites after refinement will be reported; using bitwise operations on the raw syllable values, we can refine the single-site resolution boundaries for the $\tilde{c}$ potential long matches in $O(\beta \tilde{c})$ time (see Supplementary Appendix S5.3 for details).

In Syllable-Query, we search for ongoing long matches, as opposed to past solutions’ focus on terminated matches, due to the chaotic behavior upon match termination of general sequences in reverse prefix order. The definition of the positional prefix array guarantees that the sequences with the longest ongoing matches with a sequence $y_{i}^{k}$ at syllable *k* occur in a contiguous block around position *i* in *a_k_*. Thus, at syllable *k*, we can iterate upwards and downwards within *a_k_* starting from *t_k_* until no more long matches are available. Since the process for finding matches above *z* is analogous to that below *z*, we will only describe the process for finding matches above *z* with the implication that a similar process is performed for matches below *z* (‘above’ and ‘below’ refer to relative positions in the positional prefix array, with position 0 at the top and position *M* at the bottom).

To search for matches above *z*, we maintain a pointer *p* in *a_k_*. When there are no ongoing matches above *z*, we set $p=t_{k}-1$, and every time a match above *z* is found, *p* is decremented. We check for a match between *z* and $y_{p}^{k}$ by checking whether $h_{M}[k-l,k)=h_{a_{k}[p]}[k-l,k)$. Once this is false, there are no more matches above *z* to be found as of the current syllable *k*. Alternatively, we can cut back on the number of hash comparisons by binary searching for the final value of *p* (i.e. scouting out the block of new matches) before linearly iterating through the matches.

#### 2.4.3 Avoiding redundant counting of matches

From the process for identifying matches described above, it is evident that a match spanning *s *>* l* syllables will be counted s−l+1 times, since that is the number of syllables *k* for which the matching sequence and *z* will match over syllables [k−l,k). If a query yields matches with an average length significantly greater than the minimum length *l*, then the runtime would suffer. Thus, we seek to count every match exactly once.

Lemma 1. *Once a match with sequence x_i_ is identified immediately above z, sequence x_i_ must remain immediately above z until the match ends.*

Using Lemma 1 (see Supplementary Appendix S5.4 for the proof), we can avoid redundantly visiting a match immediately above *z* at every syllable k<m≤k−l+s, after identifying it for the first time at syllable *k*, by preemptively setting our pointer *p* to $t_{m}-2$ rather than reconsidering the match. We further observe that Lemma 1 and its accompanying optimization can be generalized to any number of ongoing matches above *z*. That is, we maintain a running counter *up_on_*, and at every syllable *k*, we set $p=t_{k}-1-up_{on}$ and every time another match is identified above *z*, we decrement *p* and increment *up_on_*, thereby bypassing previously identified matches.

**Fig. 2.**
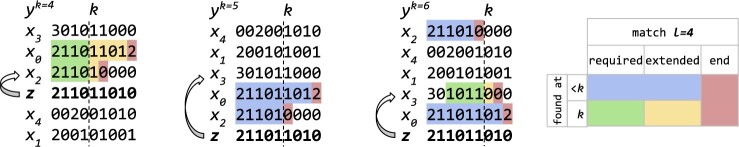
The process of finding long matches. The states of the algorithm at *k *=* *4, 5, 6 are shown. The arrows indicate setting the pointer *p* to skip over the previously found matches. At *k *=* *4, no matches have been previously found, so *p* is set to the sequence immediately above *z*, and the block of two matches above *z* are found. At *k *=* *5, the two previously found matches are skipped, but no new matches are found. At *k *=* *6, one of the previously found matches has terminated, so we skip over the remaining ongoing match to find the new match

The remaining task is to decrement *up_on_* every time we reach the end of a previously ongoing match. Let us maintain an array *up_end_* such that $up_{\mathrm{end}}[k]$ stores the number of ongoing matches that end at syllable *k*, so that we can reduce *up_on_* by $up_{\mathrm{end}}[k]$ before looking for matches at syllable *k*. To keep *up_end_* updated, we must find the total match length *s* of every match we identify and increment $up_{\mathrm{end}}[k-l+s]$. To do so efficiently, we binary search for the end of the match, checking whether $h_{M}[k,m)=h_{a_{k}[p]}[k,m)$ to test if a syllable *m* is a valid match end. Figure 2 summarizes the process for finding long matches. Since there are *n* syllables over which we potentially must binary search, the runtime of extending the $\tilde{c}$ potential matches is $O(\tilde{c}\;\text{log}\;n)$.

In genetic sequence data, recombination events result in match lengths of non-uniform distribution. To take advantage of the disproportionately large number of relatively short matches, we formulate the following heuristic: we begin by linear searching for the match end using the syllabic panel for several iterations (e.g. 10, covering 10*B* sites). If our match is among the few exceptionally long ones, we then switch to binary search with hashes for the remaining syllables. This way, we are able to find the match end in a small constant time without hashing in the average case, while bounding the runtime by O(log n) in the worst case.

#### 2.4.4 Allowing for queries in panels with unevenly distributed sites

When querying with genetic distance (cM) or physical distance (bps), site locations are non-decreasing but not necessarily uniformly distributed. With proper bookkeeping and traversal, we can query with unevenly distributed sites without affecting the time or space complexity. For the sake of brevity, refer to our code for details.

## 3 Results

We benchmarked Syllable-Query using *B *=* *64 and *B *=* *128 for reasons discussed in Supplementary Appendix S5.1. For comparison, we refer to Algorithm 3 of Sanaullah *et al.* (2021) as the full memory algorithm. We chose this algorithm because it is the most time- and space-efficient previously published solution to the *L*-long match query problem, according to the theoretical and empirical evidences in Sanaullah *et al.* (2021). Between the static and dynamic versions of the algorithms presented with d-PBWT, we chose to implement the static version of the full memory algorithm for consistency with the static nature of Syllable-Query and because the static version is more competitive in terms of memory. Sanaullah *et al.* (2021) also presented Algorithm 4, which solves the same problem, but we exclude it from our benchmarks because their benchmarks show that its runtime is not notably different from that of Algorithm 3.

**Fig. 3.**
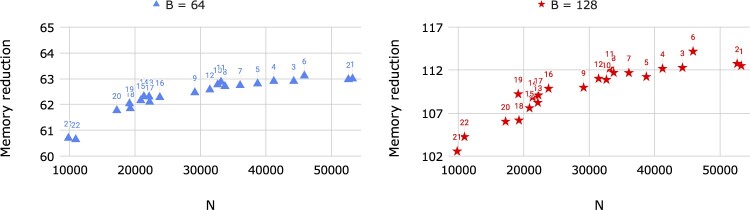
Memory reduction factors of Syllable-Query compared to the full memory algorithm versus the number of sites *N* over the UK Biobank autosomal chromosome genotype data (*M *=* *974 818). Each dot is labeled with its chromosome number

We observed the full memory usage on chromosome 21 (9793 sites) and chromosome 17 (22 215 sites) from UK Biobank (974 818 haplotypes) to be 150.4 GB and 341.2 GB. Since the asymptotic memory usage of the full memory algorithm is known to be proportional to the panel size *MN*, we extrapolated the full memory requirement for querying on the 22 autosomal chromosomes from UK Biobank consisting of 974 818 haplotypes and 658 720 markers to be 10.1 TB. In comparison, Syllable-Query used only 162 GB and 91.4 GB with *B *=* *64 and *B *=* *128, respectively, for the same task, yielding respective memory reduction factors of 62 and 110. Figure 3 provides the memory usage reductions for every chromosome based on its size. The positive trend between memory reduction and the number of sites is due to the positive trend between the number of sites and marker density per genetic distance, allowing the syllable dictionaries *r* to use less space per syllable. Over the 22 autosomal chromosomes collectively, we observed ρ≈28.3 for *B *=* *64 and ρ≈7.2 for *B *=* *128, demonstrating that *ρ*, which is inversely proportional to the space taken by our dictionaries *r*, is likely to be of considerable magnitude for genetic sequence data due to linkage disequilibrium.

We benchmarked the runtime of Syllable-Query with respect to the number of matches, sites, and haplotypes on data from UK Biobank and the 1000 Genomes Project. In every panel, we randomly removed 50 individuals to use for our 100 query haplotypes and recorded the average CPU runtime (on a single core of an AMD EPYC 7763 64-Core Processor) and the average number of matches over the 100 queries. Our intention behind running many queries in succession was to stabilize the degree of runtime volatility due to factors such as the CPU cache, as well as to simulate the practical setting of matching a query panel against a predefined panel.

**Fig. 4.**
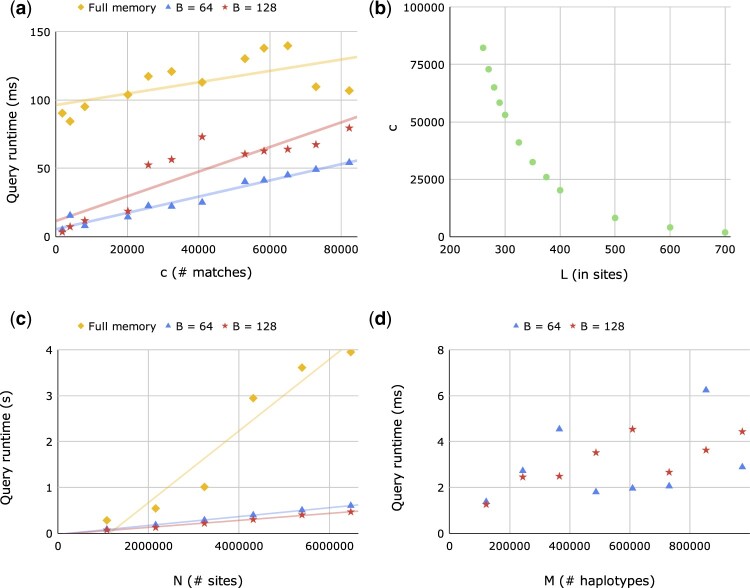
Benchmarking on UK Biobank and 1000 Genomes data. (**a**) The average query runtime versus the average number of matches *c* for each algorithm on chromosome 16 of UK Biobank (M=974,718;N=23,774). (**b**) The average number of matches *c* versus the minimum match length *L*, where the *x*-axis of part (a) corresponds to the *y*-axis of part (b). (**c**) Average query runtime versus *N* on chromosome 1 sequence data from the 1000 Genomes Project (*M *=* *4908). *N* was varied by choosing roughly uniformly distributed subsets of sites. (**d**) Average query runtime versus *M* on chromosome 1 of UK Biobank (*N *=* *53 260). (Due to the high memory demands of the full memory algorithm, we ran it only for the first 4 data points; however, for readability they are not shown, since each of its runtimes is more than 50 times either one of the corresponding Syllable-Query runtimes.)

Figure 4a shows the runtime of Syllable-Query to scale about linearly with the number of matches *c* and puts it into perspective with the full memory algorithm runtime. The most observable increases in the Syllable-Query runtime trend occur when *L* drops below *kB −* 1 for some small integer *k* (see Supplementary Appendix S5.5 for why). Figure 4b motivates our match extension heuristic by confirming the relative shortness of the average match length. Moreover, the comparable slopes of the trendlines in Figure 4a demonstrate that our virtual insertion and match extension heuristics are satisfactorily fast for real data (recall that the full memory algorithm is known to scale very well with the number of matches as it processes matches upon termination only). The *y*-intercepts of the trendlines further reveal that Syllable-Query is significantly faster than the full memory algorithm, even in the computationally unfavorable situations with small *L* mentioned above. We attribute the speedup in performance primarily to two reasons: (i) with *B* times fewer syllables than sites, the syllabic panel is much faster to iterate through. Although the reduced number of syllables is accompanied by a slight runtime factor *β* mentioned in Supplementary Appendix S5.1, *β* only appears in the match refinement stage and therefore minimally affects runtime, evident in the only slightly higher slope of the *B *=* *128 trendline than that of *B *=* *64 in Figure 4a. (ii) The CPU cache grants the CPU fast access to frequently used memory locations. Therefore, with less memory required by Syllable-Query, the CPU cache loads in data pertaining to more sites at a time, thereby saving time on data retrieval.

Figure 4c confirms the roughly linear runtime of Syllable-Query with respect to *N*. The memory usage for the benchmark on sequence data depicted in Figure 4c (M=4,908;N=6,468,094) using *B *=* *64 and *B *=* *128 was, respectively, 7.9 GB (ρ≈61) and 4.1 GB (ρ≈28), as opposed to an extrapolated 500 GB required by the full memory algorithm. The significantly higher *ρ* values compared to those for the UK Biobank autosomal genotype data are to be expected, since genotype data, unlike sequence data, are limited to genetic variants, giving rise to more distinct syllable values. Figure 4d shows a diminishingly positive correlation between query runtime and *M*, as to be expected by the log M factor involved in virtual insertion; note too that another cause of the positive correlation is the more effective CPU cache usage for smaller *M*. Due to the term log M, as *M* approaches infinity, our time complexity (but not the full memory algorithm’s) approaches infinity, but our benchmarking reveals this growth rate to be negligible in practice.

To verify the empirical correctness of Syllable-Query beyond the prior theoretical discussion, especially concerning the reliability of our hash function, we ran 1000 distinct queries each for *L* in terms of sites and cM on UK Biobank data, totaling over one million matches. For every query, Syllable-Query reported the same matches as the full memory algorithm.

For genealogical search, query lengths of at least 5 or 7 cM and 700 single nucleotide polymorphisms (SNPs) are typically chosen, as established by simulations conducted by the DTC company 23andMe (23andMe, 2021; Roberts *et al.*, 2011). Despite the minimum query length required by Syllable-Query (see Supplementary Appendix S5.1), we found that our site and cM requirements using deCODE genetic maps were well below these cutoffs (see Supplementary Appendix S5.6), so our requirements would not limit the application of Syllable-Query to genealogical search.

## 4 Discussion and conclusions

We have presented the Syllable-PBWT framework as a space-efficient alternative to the conventional binary PBWT. The main methodological contribution of this work is the redesign of the PBWT long match query algorithm by stripping away the most memory-intensive PBWT data structures. Transforming the binary panel into a syllabic panel can be viewed as abstracting away fine detail to lighten the memory load while retaining the information required for finding long matches. Most importantly, we introduce hash arrays to underpin Syllable-Query’s ability to query without the full-panel-sized arrays *d*, *u*, *v*. Although using *d*, *u*, *v* in a transient fashion for all-vs-all matching is appropriate, making them persistent for one-vs-all query is overly memory-costly. With the hash arrays *h*, we maintain the constant runtime exhibited by *d* for checking whether a match is long enough. Moreover, *h* can substitute *u* and *v* for virtual insertion but with the incurrence of a small worst-case O(log M log n) runtime factor for binary search.

While in this article, we aimed to present the most space-efficient solution by putting all the above design elements together, it is worth reviewing their individual contributions. The biggest memory reduction comes from creating the syllabic panel and replacing the full-panel-sized a,d,u,v with syllabic versions of *a* and *h*. To reduce the size of the panel itself, we use coordinate compression to bring the overall memory reduction factor to about *B*. If memory efficiency is not the sole priority, one may mix-and-match the design elements to create simpler alternative algorithms with lower degrees of space efficiency.

Although the primary goal of this work is to reduce the memory footprint of the long match query algorithms, some elements of our algorithms can be used for other purposes. For example, the coordinate compression in Syllable-PBWT can be a solution for lossless compression of the PBWT panel. Unlike run-length compression of the divergence array mentioned by Durbin (2014) which is not friendly for real-time querying, our Syllable-PBWT data structures support regular PBWT algorithms within the compressed format without decompression. Therefore, our algorithm can also be applied to all-vs-all matching; a naive method is to query each haplotype against the rest, although there may be a more efficient method.

Conceptually, Syllable-PBWT is reminiscent of multiallelic-PBWT (mPBWT) (Naseri *et al.*, 2019b) in that the sequence values are elements of a variably-sized alphabet. However, Naseri *et al.* (2019b) only presented algorithms for panel construction and all-vs-all matching in multiallelic data but none for one-vs-all query. A contribution of this work is the long match query algorithm absent from mPBWT. Of note, the Graph BWT (GBWT) is another multi-allelic extension of PBWT (Sirén *et al.*, 2019). GBWT builds a haplotype index in the space of a graph rather than linear sequences. While GBWT is more general, it is also quite heavy. There is a potential application of the syllable compression concept to GBWT.

Please note that for applications such as phasing and imputation (Delaneau *et al.*, 2019; Rubinacci *et al.*, 2020) when searching for informative conditioning states, high *B* values such as 128 will result in missing short matches, especially in low SNP density array data. In such cases, it would be preferable to use a smaller *B*. Assuming a marker density of one site on average in every 3.5 kb, if *B *=* *128, then the minimum cutoff for finding all matches should be at least 255 sites, corresponding to ∼0.893 Mb. For *B *=* *64, the minimum cutoff will be ∼0.445 Mb.

Despite the utility of *L*-long matches, one drawback is their requirement for match exactness, whereas real data often contain genotyping and phasing errors. Encouragingly, the contributions in this work could be adapted to a mismatch-tolerant variation of the *L*-long match query problem. Since past efficient solutions only consider matches upon termination, little potential remains for looking past match interruptions. In Syllable-Query, on the other hand, matches are considered as soon as they reach the threshold length and are then manually extended. Therefore, the extension process can be modified to continue as long as the number of mismatches remains below a specified parameter. The various starts and ends of the fragmented match could then be recorded in an event schedule, a more intricate development of the match end tracker in our current algorithm, to swiftly bypass the fragments composing previously found matches.

Beyond methodological contributions, we showed that Syllable-PBWT and Syllable-Query delivered a memory reduction factor of over 100 in real sequences from the 1000 Genomes Project and the UK Biobank. For UK Biobank, while the state-of-the-art query algorithm (Sanaullah *et al.*, 2021) requires 10 TB of memory, Syllable-Query only requires 91 GB. This innovation will allow online genealogical search to be conducted with much more modest hardware and on even larger data sets in the future.

## Supplementary Material

btac734_Supplementary_Data
