# Supplementary material for: Syllable-PBWT for space-efficient haplotype long-match query
Source: Bioinformatics. 2022 Nov 28;39(1):btac734. doi: 10.1093/bioinformatics/btac734 (PMC9805553; doi:10.1093/bioinformatics/btac734)
Supplement: btac734_Supplementary_Data [file btac734_supplementary_data.pdf]

SYLLABLE-PBWT FOR SPACE-EFFICIENT HAPLOTYPE LONG-MATCH QUERY

Victor Wang, Ardalan Naseri, Shaojie Zhang and Degui Zhi

1 APPENDIX

1.1 Choice of B

Considering the factors below, we implement Syllable-Query with $B \in \{64, 128\}$.

- The value of B restricts the smallest allowable query length because if a small query length L causes $l = 0$, then the runtime would skyrocket. If we were to override l to be 1, then matches that do not extend over at least one full syllable would pass unseen by our algorithm. Specifically, in the worst case scenario, a match may nearly entirely span two adjacent syllables ($2B - 2$ sites), but we would be unable to detect it. If, however, the match is any longer, it must span at least one full syllable, meaning that $2B - 1$ sites is the smallest query length that a given B can handle. Letting B be 64 or 128 leads the smallest query length to be 127 or 255 sites, respectively, which are well below the query lengths used to detect IBD segments in practice. Similarly, for variably distributed sites (i.e. when querying with genetic or physical distance), the query length must be greater than the longest distance spanning exactly two adjacent syllables. See Appendix 1.6 for evidence of these B values yielding genetic query length requirements that comfortably allow for IBD segment detection. Further increasing B would be accompanied by the risk of limiting practical use.
- In order to store and operate on the raw syllable values, the programming language we use for implementation must provide data types to support a B-bit integer. The GNU C++ compiler provides the `unsigned long long` data type which supports $B = 64$, and on 64-bit processors, it also provides the `unsigned __int128` data type which supports $B = 128$. These built-in data types motivated our use of these B values, although the Boost C++ libraries could technically support even higher values of B.
- The predominance of 64-bit processors in recent decades has allowed for typical instructions involving 64-bit data to be executed within a single CPU clock cycle (Hunt, 1995; Kohn and Margulis, 1989). Choosing $B = 64$ would allow us to make most efficient use of this architecture, while choosing $B = 128$ would introduce a small constant runtime factor to bitwise operations on raw syllable values. In general, the runtime factor of basic operations on raw syllable values for a chosen B is $\beta \approx \lceil \frac{B}{64} \rceil$, because while instructions involving B-bit data ($B > 64$) do not necessarily require multiple clock cycles, such is the worst case scenario.
- The final motivation for not further increasing B, even though doing so would lead to more memory reduction, is the diminishing rate at which increasing B increases the memory reduction factor. The rate is diminishing because as B increases, raw syllable values are more likely to be distinct, so the ratio ρ will decrease, causing the memory usage of r to decrease evermore slowly as B increases.

1.2 Reliability of the polynomial hash function

To create a strong hash function with minimal chance of collisions, several conditions must be satisfied: the modulus should be a large prime, the polynomial variable should be greater than the largest value in the input sequence, and the modulus and polynomial variable should be relatively prime (Alomair *et al.*, 2010; Karp and Rabin, 1987). In our implementation, we set $BASE = 2^{48} - 59$ and $MOD = 2^{64} - 59$, satisfying these criteria (as well as bounding every hash to fit within a 64-bit integer), so our polynomial hash function can be considered a pseudorandom function through which two identical inputs are guaranteed to yield the same hash and two distinct inputs have a $p = \frac{1}{MOD}$ chance to yield the same hash. To illustrate the theoretical robustness of the hash function, suppose that we were to perform $q = 10^{10}$ independent comparisons of distinct sequences. The chance $P(p, q) = 1 - (1 - p)^q$ of a positive number of collisions occurring and the expected number $E(p, q) = pq$ of collisions would both be less than 10^{-9}, demonstrating that even with a number of distinct-sequence comparisons that dwarfs what would be required in a practical setting, the expected chance of a false positive remains negligible.

1.3 Refining match boundaries

To obtain the single-site resolution of the match boundaries in $O(\beta)$ time, we perform bitwise operations on the raw syllable values where a match (non-inclusively) starts and ends. The GNU C++ compiler provides the bitwise XOR operator ^ (which gives an integer in which every bit is 0 for which the two operands have the same value) and the functions `__builtin_clzll` and `__builtin_ctzll` which count the number of leading and trailing 0s, respectively, in a variable of the 64-bit data type `unsigned long long`. These functions can be used to count the number of leading or trailing 0s in the bitwise XOR between two integers, giving the length of the suffix or prefix match, respectively, of the reverse binary representations of the integers (recall that the raw syllable values are reversed binary haplotype substrings parsed as binary numbers). For $B = 128$, we see β come into play because the left and right 64-bit halves of the 128-bit integer have to be operated on separately, so the runtime of refining the $\tilde{c}$ potential long matches is $O(\beta\tilde{c})$.

1.4 Proof of Lemma 1

PROOF. In Algorithm 2 of PBWT, Durbin observes that the start b of the longest ongoing match at site k between y_i^k and $y_{j>i}^k$ is $\max_{i<m\leq j} d_k[m]$, where the divergence array d_k is defined such that $d_k[0] = k$, and for $i > 0$, $d_k[i]$ is the smallest b for which $y_{i-1}^k[b, k) = y_i^k[b, k)$. With this observation, $y_{t_k-1}^k$ must be the sequence with which (1) z has the longest ongoing match, (2) among sequences with a reverse prefix over syllables $[0, k)$ that is lexicographically less than that of z. Condition (1) must be satisfied because no other match above z can spontaneously obtain an earlier start, which by definition is not earlier than that of the match with sequence x_i. Condition (2) must be satisfied because the match persisting as k increases implies that the same values are being prepended to the reverse prefixes of z and x_i, thereby not affecting their lexicographical ordering. Even if sequence x_i has an equally long match with z as another sequence x_j above z, the construction of the positional prefix array based off that of the previous syllable guarantees the retention of the relative positions of sequences between which a match persists. Therefore, until the match ends, both conditions will remain satisfied, implying that $a_m[t_m - 1] = i$ at every syllable $k \leq m \leq k - l + s$.

1.5 Syllable-Query runtime on small L

To see why the most observable increases in the Syllable-Query runtime occur when L drops below $kB - 1$ for some small integer k, recall that for Syllable-Query to identify all matches spanning L sites, it must consider all $\tilde{c}$ potential long matches spanning $l = \lfloor \frac{L-B+1}{B} \rfloor$ syllables, which means that, for example, when $L < 3B - 1$, matches spanning a mere syllable must be considered, whereas when $L = 3B - 1$, only matches spanning at least 2 syllables are potential matches. These runtime spikes are only expected to be considerable for relatively small L, since genetic recombination leads the absolute rate of change of c with respect to L to start large before drastically dropping off, as shown in Figure 4b.

1.6 Syllable-Query length requirements

To confirm the practicality of Syllable-Query despite the restriction on query length (see Appendix 1.1 for details), we measured the smallest queryable genetic length (SQGL) in cM for each autosomal chromosome with the deCODE genetic map (Halldorsson *et al.*, 2019) in UK Biobank data. For $B = 64$ and $B = 128$, the smallest queryable site lengths are 127 and 255, respectively. The highest SQGL for 127 and 255 sites was 2.7 and 4.2 cM, respectively. The average SQGL for 127 and 255 sites was 2.2 and 3.3 cM, respectively. These requirements are well below the query lengths of 5 or 7 cM and 700 SNPs utilized by 23andMe for genealogical search (23andMe, 2022; Roberts *et al.*, 2011). Since UK Biobank has a relatively low marker density, our SQGLs would likely also support genealogical search on biobanks in general. Unless especially small L values are to be used, we recommend choosing $B = 128$ for greater memory reduction, given the similar runtimes between $B = 64$ and $B = 128$ shown in Figure 4.

REFERENCES

23andMe (2022). DNA Relatives: Detecting Relatives and Predicting Relationships. `https://customercare.23andme.com/hc/en-us/articles/212170958-DNA-Relatives-Detecting-Relatives-and-Predicting-Relationships`.

Alomair, B., Clark, A., and Poovendran, R. (2010). The power of primes: security of authentication based on a universal hash-function family. *Journal of Mathematical Cryptology*, **4**(2), 121–148.

Halldorsson, B. V., Palsson, G., Stefansson, O. A., Jonsson, H., Hardarson, M. T., Eggertsson, H. P., Gunnarsson, B., Oddsson, A., Halldorsson, G. H., Zink, F., Gudjonsson, S. A., Frigge, M. L., Thorleifsson, G., Sigurdsson, A., Stacey, S. N., Sulem, P., Masson, G., Helgason, A., Gudbjartsson, D. F., Thorsteinsdottir, U., and Stefansson, K. (2019). Characterizing mutagenic effects of recombination through a sequence-level genetic map. *Science*, **363**(6425).

Hunt, D. (1995). Advanced performance features of the 64-bit pa-8000. In *Digest of Papers. COMPCON'95. Technologies for the Information Superhighway*, pages 123–128. IEEE.

Karp, R. M. and Rabin, M. O. (1987). Efficient randomized pattern-matching algorithms. *IBM Journal of Research and Development*, **31**(2), 249–260.

Kohn, L. and Margulis, N. (1989). Introducing the intel i860 64-bit microprocessor. *IEEE Micro*, **9**(4), 15–30.

Roberts, M. E., Riegert-Johnson, D. L., and Thomas, B. C. (2011). Self Diagnosis of Lynch Syndrome Using Direct to Consumer Genetic Testing: A Case Study. *Journal of Genetic Counseling*, **20**(4), 327–329.
